# The Synthesis and the Biological Evaluation of New Thiazolidin-4-one Derivatives Containing a Xanthine Moiety

**DOI:** 10.3390/molecules18089684

**Published:** 2013-08-13

**Authors:** Florentina Geanina Lupascu, Oana Maria Dragostin, Liliana Foia, Dan Lupascu, Lenuta Profire

**Affiliations:** Department of Pharmaceutical Chemistry, Faculty of Pharmacy, “Grigore T. Popa” University of Medicine and Pharmacy, 16 University Street, Iasi 700115, Romania; E-Mails: geani0407@yahoo.com (F.G.L.); farmacist_oanamaria@yahoo.com (O.M.D.); Department of Biochemistry, Faculty of Dentistry, “Grigore T. Popa” University of Medicine and Pharmacy, 16 University Street, Iasi 700115, Romania; E-Mail: lilifoia@yahoo.co.uk

**Keywords:** thiazolidin-4-one, xanthine, synthesis, spectral methods, antioxidant effect

## Abstract

Starting from theophylline (1,3-dimethylxanthine) new thiazolidin-4-one derivatives **7a_1–7_**, **7b_1–7_** have been synthesized as potential antidiabetic drugs. The structure of the new derivatives was confirmed using spectral methods (FT-IR, ^1^H-NMR, ^13^C-NMR). The *in vitro* antioxidant potential of the synthesized compounds was evaluated according to the ferric reducing power, the total antioxidant activity and the DPPH and ABTS radical scavenging assays. Reactive oxygen species (ROS) and free radicals are considered to be implicated in a variety of pathological events, such as diabetes mellitus and its micro- and macrovascular complications. The results of chemical modulation of the thiazolidin-4-one intermediaries **6a**, **6b** through condensation with several aromatic aldehydes is the improvement of the antioxidant effect. All benzylidenethiazolidin-4-one derivatives **7a_1–7_**, **7b_1–7_** are more active than their parent thiazolidin-4-ones. The most active compounds are the ones obtained by reaction of condensation with 4-hydroxybenzaldehyde (compounds **7a_5_**, **7a_6_**), 4-dimethylaminobenzaldehyde (compounds **7a_6_**, **7b_6_**) and 2-nitrobenzaldehyde (compounds **7a_7_**, **7b_7_**).

## 1. Introduction

**Figure 1:**
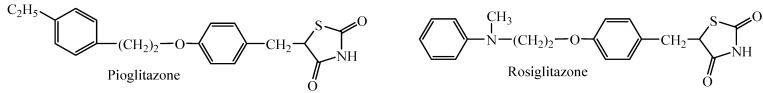
The structure of pioglitazone and rosiglitazone.

The thiazolidine ring system represents a very important structural unit in drug discovery [1]. A survey of the literature reveals that extensive studies have been carried out on the synthesis of thiazolidines. These compounds are known to exhibit interesting biological activity such as antimicrobial [2,3,4,5], antitubercular [6], antipsihotic [7], anticancer [8,9], antiviral [10], anti-HIV [11], anti-inflammatory [12,13] and antihyperglycemic [14,15,16,17] effects. As antidiabetic drugs, thiazolidinediones (pioglitazone—Actos^®^ and rosiglitazone—Avandia^®^, Figure 1) have an important place in type 2 diabetes mellitus therapy [18].

Diabetes mellitus, a chronic metabolic disorder, is a major health problem that is approaching pandemic proportions worldwide. It is characterized by absolute or relative deficiencies in insulin secretion and/or insulin action [19]. An inadequate glycaemic control can be the leading cause of cardiovascular disorders, blindness, renal failure and amputations [19,20]. Type 2 diabetes mellitus (T2DM) accounts for over 90% of the disease cases and it is characterized by high levels of glucose resulting from progressive pancreatic β-cell dysfunction caused by chronic insulin resistance [19]. As agonists of nuclear receptor peroxisome proliferator-activated receptor gamma (PPAR-γ), thiazolidinediones (TZD) reduce insulin resistance in the liver and peripheral tissues; increase the intake of insulin-dependent glucose and decrease withdrawal of glucose from the liver [14,18]. On the other hand, the thiazolidine derivatives have been reported to have antioxidant activities that could be effective in the T2DM therapy [21,22,23].

A relationship between reactive oxygen species and many diseases including diabetes, myocardial infarction, neurological disorders, asthma, cancer, rheumatoid arthritis has been observed [24], so in case of diabetic patients, elevated levels of blood glucose is the main cause which leads to an increased production of free radicals, especially reactive oxygen species resulted from glucose auto-oxidation, lipid peroxidation and protein glycosylation. Endothelial cells chronically exposed to oxidative stress lead to diabetes long term complications (micro- and macrovascular) [19]. 

**Figure 2:**
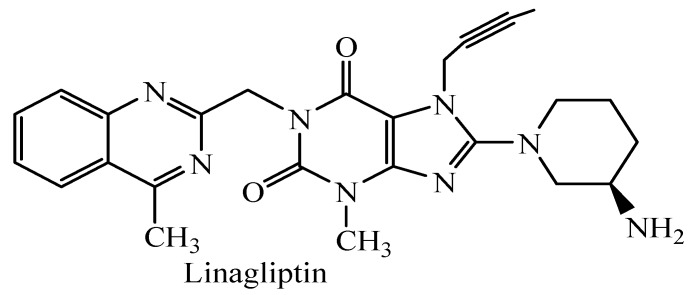
The structure of linagliptin.

The latest research regarding antidiabetic drugs has focused on dipeptidyl peptidase IV inhibitors that act by increasing the concentrations of the endogenous incretins GLP-1 and GI, and consequently reducing of fasting post-prandial hyperglycaemia [25]. The most recently marketed inhibitor is linagliptin (Ondero^®^, Trajenta^®^, Figure 2), a nonpetidommimetic drug with a xanthine structure, which received approval in 2011 [26]. At the same time xanthine derivatives are known to have antioxidant and radical scavenging activity that contributes to their pharmacological properties [27,28].

Based on the arguments presented above, we are reporting here the design, synthesis and antioxidant effects of new heterocyclic compounds which combine a thiazolidine structure with a xanthine one, as potential antidiabetic agents.

## 2. Results and Discussion

### 2.1. Chemistry

**Scheme 1:**
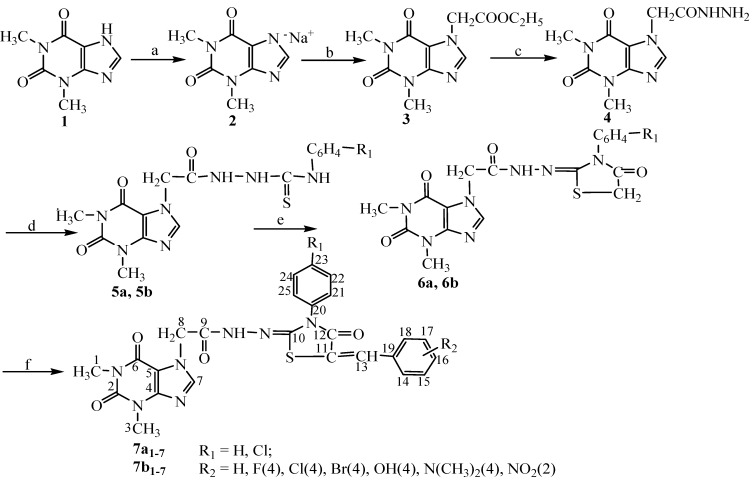
Synthetic procedure for compounds **7a_1–7_**, **7b_1–7_**.

The thiazolidine derivatives **7a_1–7_**; **7b_1–7_** were prepared using the method summarized in Scheme 1.

*Reagents and conditions*: (**a**) sodium, absolute methanol; (**b**) ethyl chloroacetate, ethanol:DMFA, heating 7 h; (**c**) hydrazine hydrate, ethanol, heating 6 h; (**d**) phenyl isothiocyanate/4-chlorophenyl isothiocyanate, 1,4-dioxane:DMFA, heating 6 h; (**e**) chloroacetyl chloride, methanol:chloroform, heating 10 h; (**f**) aromatic aldehydes, 1,4-dioxane, piperidine, heating 6 h.

Through the esterification of theophylline (1,3-dimethylxanthine, **1**) sodium salt **2** with ethylcloroacetate, (1,3-dimethylxanthin-7-yl)ethyl acetate (**3**) was obtained, which was turned into hydrazide derivative **4** [29]. During the next step this intermediate was reacted with phenylisothiocyanate and 4-chlorophenylisothiocyanate leading to the corresponding thiosemicarbazide derivatives **5a**, **5b**. By cyclization of **5a**, **5b** with chloracetyl chloride the corresponding thiazolidin-4-one derivatives **6a**, **6b** were obtained. The last step of the synthesis consisted in the condensation of the thiazolidin-4-one derivatives with several aromatic aldehydes—benzaldehyde, 4-bromo-/4-fluoro-/4-chloro-/4-hydroxy-/4-dimethylamino-/2-nitrobenzaldehyde.

The structure of the compounds was assigned on the basis of spectral data (IR, ^1^H-NMR, ^13^C-NMR). In the IR spectra of the thiosemicarbazide derivatives 5a, 5b the characteristic absorption band of the thione group (C=S) was observed at 1160 cm^−1^ (**5a**), and 1188 cm^−1^ (**5b**), respectively. The valence vibration of the bond C-Cl (**5b**) produced a narrow absorption band of medium intensity at 825 cm^−1^. In the ^1^H-NMR spectra of the compounds the aromatic protons resonated as multiplets in the 7.19–7.47 ppm region (**5a**), or the 7.31–7.51 ppm region (**5b**), respectively, confirming the success of the reaction between hydrazide derivative **4** and the phenyl isothiocyanates. The protons of the xanthine structure were also identified in the spectra of the thiosemicarbazides. The protons of the two methyl groups appeared as two singlets at 3.09 ppm and 3.45 ppm (**5a**), or at 3.22 ppm and 3.57 ppm (**5b**), respectively. The proton of the methine group resonated as a singlet at 8.09 ppm, while the protons of the methylene group also resonated as a singlet in the 5.08–5.09 ppm region.

In the IR spectra of thiazolidin-4-ones derivatives **6a**, **6b** the characteristic band of the thione group (C=S) disappeared and the appearance of the C-S bond characteristic of the thiazolidin-4-one it was observed in the range of 690–695 cm^−1^. The ^1^H-NMR spectra of the compounds showed the methylene protons of the thiazolidin-4-one ring at 3.67 ppm (**6a**) and at 3.78 ppm (**6b**) resonating as a singlet. That confirmed the formation of the thiazolidin-4-one ring.

The structure of the benzylidenethiazolidin-4-one derivatives **7a_1–7_**, **7b_1–7_** was confirmed by the appearance in the ^1^H-NMR spectra of the proton signal characteristic for the methine group from the benzilidene thiazolidin-4-one ring as a sing let in the range of 7.28–7.38 ppm (**7a_1–7_**) and at 7.24–7.51 ppm (**7b_1–7_**). In the ^13^C-NMR spectra the characteristic signal for this methine group appeared in the 140.35–143.07 ppm range (**7a_1–7_**), or 138.51–142.84 (**7b_1–7_**), respectively.

### 2.2. Biological Evaluation

#### 2.2.1. Ferric Reducing Power

The measurement of the reducing power defines an important aspect of the compounds’ antioxidant activity. In the presence of the electron-donating compounds, the potassium ferric/ferricyanide complex is reduced to its ferrous form (Fe^2+^). The amount of Fe^2+^ is then quantitatively monitored by measuring the intensity of the Perls Prussian blue colour complex formed by reaction with ferric chloride, at 700 nm [30]. The reaction between the ferrous form and the ferric chloride is:

4FeCl_3_ + 3K_4_[Fe(CN)_6_] → Fe[Fe(CN)_6_]_3_ + 12KCl

**Figure 3:**
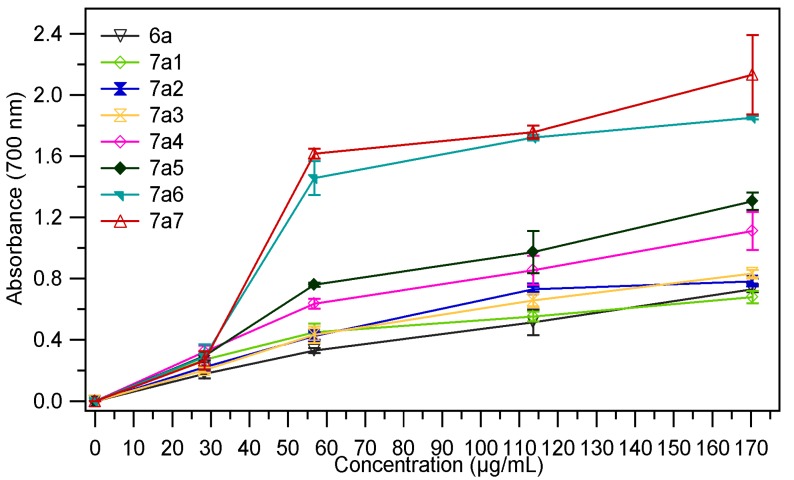
The absorbance of **7a_1–7_** in ref. to **6a**.

**Figure 4:**
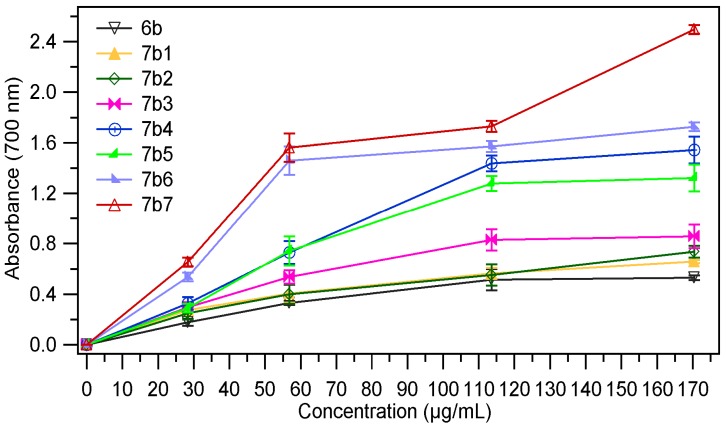
The absorbance of **7b_1–7_** in ref. to **6b**.

The absorbance value of the samples at different concentrations (28.4 µg/mL, 56.8 µg/mL, 113.63 µg/mL and 170.45 µg/mL) are presented in Figure 3, Figure 4. The results expressed as EC_50_ values (mg/mL) are shown in Table 1. Low values of EC_50_ demonstrate a higer ferric reducing power.

As we expected the absorbance of the sample increased with the concentration, thus proving the fact that reducing power of the tested compounds is concentration-dependent. The analysis of the data presented in the Table 1, revealed that the chemical modulation of the thiazolidin-4-one ring through condensation with several aromatic aldehydes had a good influence on the antioxidant potential; all tested compounds being more active than the corresponding thiazolidin-4-ones **6a**, **6b**. It was also observed that both series of compounds **7a_1–7_**, **7b_1–7_** have a comparable reducing power. In the series of 3-phenylthiazolidin-4-one derivatives **7a_1–7_** the most active compounds were those which resulted from condensation with 4-hydroxybenzaldehyde (compound **7a_5_**) and 4-dimethlaminobenzaldehyde (compound **7a_6_**). For these compounds the EC_50_ values were 0.033 ± 0.0019 (for **7a_5_)** and 0.033 ± 0.0012 (for **7a_6_**), which means that they are about four times more active than the corresponding thiazolidin-4-one (**6a**, EC_50_ = 0.137 ± 0.0036). Regarding the compounds **7b_1–7_** obtained through condensation of 3-(4-chlorophenyl)-thiazolidin-4-one derivative **6b** with several aromatic benzaldehydes, we could observe that the most active compounds were **7b_5_** and **7b_6_**, the corresponding analogues of **7a_5_** and **7a_6_**. These compounds were about 5.7 times (**7b_5_**, EC_50_ = 0.027 ± 0.0013) and 6.7 times (**7b_6_**, EC_50_ = 0.023 ± 0.0011) more active than the corresponding thiazolidin-4-one derivative (**6b**, EC_50_ = 0.155 ± 0.0038). The chemical modulation of the parent thiazolidin-4-one derivatives improved their ferric reducing power, all tested compounds being more active than 3-phenlythiazolidin-4-one, respectively the 3-(4-chlorophenyl)thiazolidin-4-one but they are less active than the ascorbic acid (AA) at the same concentration.

**Table 1:** The ferric reducing power (EC_50_ mg/mL) of the derivatives**6a**, **6b**, **7a_1–7_**, **7b_1–7_**.

Sample	EC_50_ mg/mL	Sample	EC_50_ mg/mL
**6a**	0.137 ± 0.0036	**6b**	0.155 ± 0.0038
**7a_1_**	0.109 ± 0.0014	**7b_1_**	0.109 ± 0.0045
**7a_2_**	0.097 ± 0.0018	**7b_2_**	0.102 ± 0.0024
**7a_3_**	0.055 ± 0.0036	**7b_3_**	0.046 ± 0.0015
**7a_4_**	0.096 ± 0.0023	**7b_4_**	0.066 ± 0.0017
**7a_5_**	0.033 ± 0.0019	**7b_5_**	0.027 ± 0.0013
**7a_6_**	0.033 ± 0.0012	**7b_6_**	0.023 ± 0.0011
**7a_7_**	0.058 ± 0.0041	**7b_7_**	0.041 ± 0.0010
**AA**	0.0075 ± 0.0002		

Data are mean ± SD (n = 3, *p* < 0.005).

#### 2.2.2. Total Antioxidant Activity

**Figure 5:**
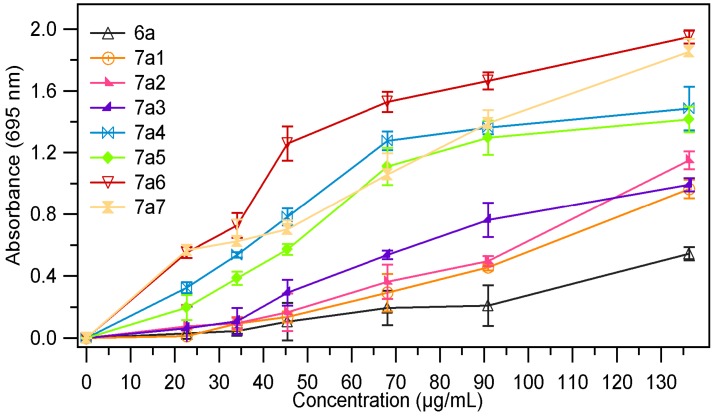
The absorbance of **7a_1–7_** in ref. to **6a**.

**Figure 6:**
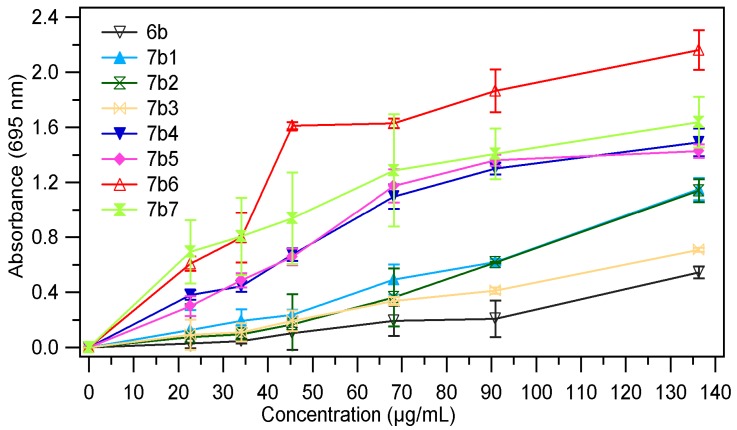
The absorbance of **7b_1–7_** in ref. to **6b**.

The total antioxidant activity was determined by the formation of phosphomolybdenum blue complex by the reduction of Mo(VI) to Mo(V) under the action of electron donating compounds. The maximum absorption of the complex was recorded at 695 nm [31]. The absorbance values of samples at different concentrations (23, 34, 45, 68, 91 and 136 µg/mL) are presented in Figure 5, Figure 6. The EC_50_ values (mg/mL) are shown in Table 2. Low values of EC_50_ indicate a higer effectiveness in antioxidant properties.

**Table 2:** The total antioxidant activity (EC_50_ mg/mL) of the derivatives **6a**, **6b**, **7a_1–7_**, **7b_1–7_**.

Sample	EC_50_ mg/mL	Sample	EC_50_ mg/mL
**6a**	0.133 ± 0.0045	**6b**	0.124 ± 0.0009
**7a_1_**	0.105 ± 0.0025	**7b_1_**	0.090 ± 0.0018
**7a_2_**	0.099 ± 0.0038	**7b_2_**	0.092 ± 0.0008
**7a_3_**	0.048 ± 0.0023	**7b_3_**	0.043 ± 0.0026
**7a_4_**	0.076 ± 0.0025	**7b_4_**	0.099 ± 0.0024
**7a_5_**	0.025 ± 0.0012	**7b_5_**	0.022 ± 0.0013
**7a_6_**	0.033 ± 0.0014	**7b_6_**	0.026 ± 0.0018
**7a_7_**	0.037 ± 0.0007	**7b_7_**	0.043 ± 0.0028
**AA**	0.0067 ± 0.0003		

Data are mean ± SD (n = 3, *p*< 0.05).

The data of this study also support the conclusion that the antioxidant activity of the tested compounds increases with concentration and that all benzylidenethiazolidin-4-one derivatives **7a_1–7_**, **7b_1–7_** are more active than parent thiazolidin-4-one derivatives (**6a**, **6b**). It was observed that the presence of chloro radical on the phenyl ring at N_3_ of thiazolidin-4-one has a good influence on the antioxidant properties, the derivatives (**7b_1–7_**) being generally more active than their analogues from the **7a_1–7_** series. In both series the most active compounds were hydroxyl- (**7a_5_**, **7b_5_)** and dimethylamino- (**7a_6_**, **7b_6_**) analogues. The hydroxyl derivatives, **7a_5_** (EC_50_ = 0.025 ± 0.0012) and **7b_5_** (EC_50_ = 0.022 ± 0.0013) were 5.3 times and 5.6 times more active than corresponding thiazolidin-4-one, **6a** (EC_50_ = 0.133 ± 0.0045), respectivelly **6b** (EC_50_ = 0.124 ± 0.0009). The antioxidant effects of the dimethylamino analogues (**7a_6_**, **7b_6_**) were similar with of hydroxyl derivatives, being 4 times (**7a_6_**, EC_50_ = 0.033 ± 0.0014), respectivelly 4.8 times (**7b_6_**, EC_50_ = 0.026 ± 0.0018), more active than parent compounds (**6a**, **6b**). The tested compounds were less active than ascorbic acid (AA) at the same concentration.

#### 2.2.3. DPPH Radical Scavenging Assay

**Figure 7:**
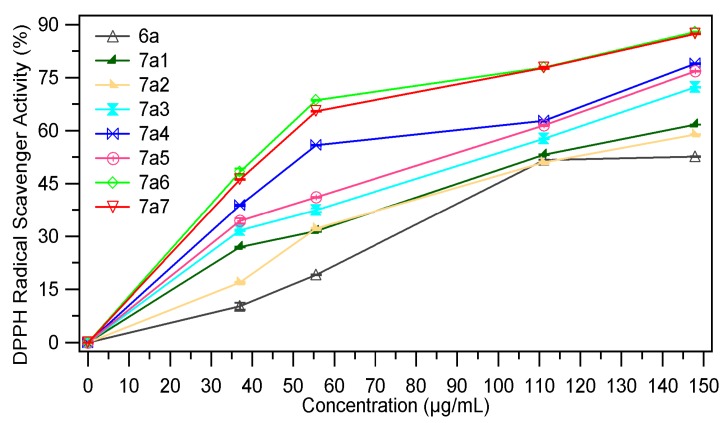
The scavenging ability (%) of **7a_1–7_** in ref. to **6a**.

**Figure 8:**
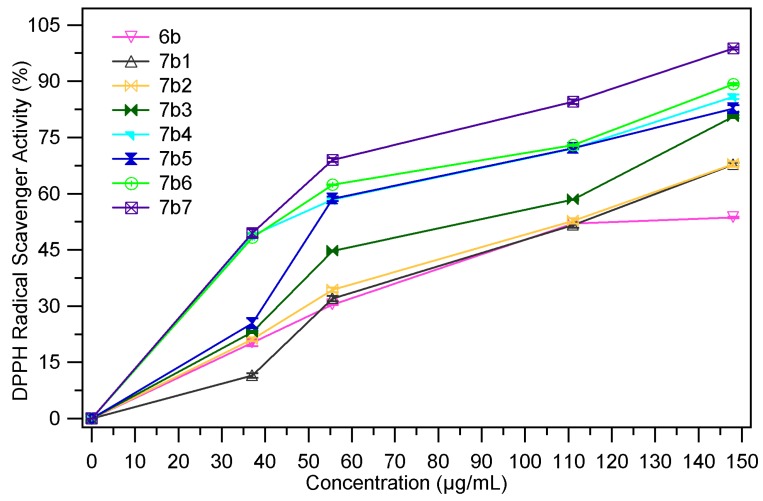
The scavenging ability (%) of **7b_1–7_** in ref. to **6b**.

DPPH (1,1-diphenyl-2-picryl-hydrazyl) is a stable free radical with a violet colour that, under the action of proton donating compounds, is reduced to a light yellow colour that this change can be monitored at 517 nm [32]. The DPPH radical scavenging ability (%) of samples at different concentrations (37 µg/mL, 55.55 µg/mL, 111.11 µg/mL and 144.80 µg/mL) are presented in Figure 7, Figure 8. The EC_50_ values (mg/mL) are shown in Table 3. Low values of EC_50_ indicate a higer effectiveness in DPPH scavenging ability.

**Table 3:** The DPPH scavenging ability (EC_50_ mg/mL) of the derivatives **6a**, **6b**, **7a_1–7_**, **7b_1–7_**.

Sample	EC_50_ mg/mL	Sample	EC_50_ mg/mL
**6a**	0.136 ± 0.0006	**6b**	0.127 ± 0.0013
**7a_1_**	0.094 ± 0.0013	**7b_1_**	0.106 ± 0.0011
**7a_2_**	0.107 ± 0.0018	**7b_2_**	0.101 ± 0.0044
**7a_3_**	0.066 ± 0.0011	**7b_3_**	0.049 ± 0.0006
**7a_4_**	0.077 ± 0.0005	**7b_4_**	0.085 ± 0.0017
**7a_5_**	0.038 ± 0.0044	**7b_5_**	0.038 ± 0.0024
**7a_6_**	0.039 ± 0.0008	**7b_6_**	0.037 ± 0.0037
**7a_7_**	0.048 ± 0.0001	**7b_7_**	0.038 ± 0.0014
**AA**	0.0065 ± 0.0031		

Data are mean ± SD (n = 3, *p*< 0.05).

The chemical modulation of the thiazolidin-4-one derivatives **6a**, **6b** through condensation with several aromatic benzaldehydes determines the improvement of the DPPH radical scavenging ability, as both series of compounds **7a_1–7_** and **7b_1–7_** were more active than their parents. In reference to compound **6a**, its benzylidene analogues **7a_1–6_** are 1.5–3.5 times more active. Under similar conditions the compounds **7b_1–7_** are slightly less active, being 1.2–3.4 times more active than the corresponding thiazolidin-4-one **6b**. The most active compounds are hydroxyl- (compounds **7a_5_**, **7b_5_**), dimethylamino- (compounds **7a_6_**, **7b_6_**) and nitro- (compounds **7a_7_**, **7b_7_**) analogues.

#### 2.2.4. ABTS Radical Scavenging Assay

**Figure 9:**
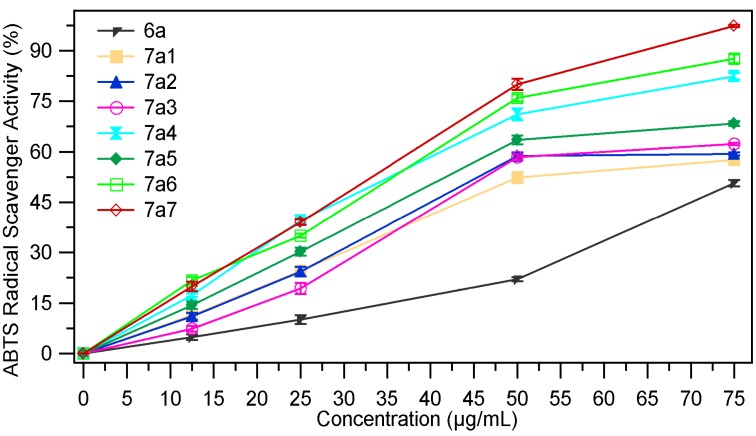
The scavenging ability (%) of **7a_1–7_** in ref. with **6a**.

**Figure 10:**
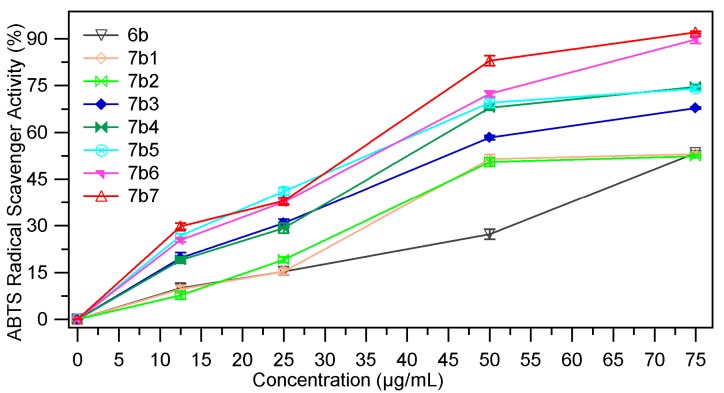
The scavenging ability (%) of **7b_1–7_** in ref. with **6b**.

The ABTS (2,2′-azino-bis(3-ethylbenzothiazoline-6-sulfonic acid)) radical cation decolourisation assay is based on the ability of the hydrogen donating antioxidants to scavenge the long-life radical cation ABTS^+^. The ABTS^+^ which is a blue chromophore is generated by the reaction between 2.2′-azino-bis(3-ethylbenzothiazoline-6-sulfonic acid) and ammonium persulfate. The antioxidant compound produces a discoloration of the solution with a decrease in the absorbance measured at 734 nm [33]. The ABTS radical scavenging ability (%) of the samples at different concentrations (12.5 µg/mL, 25 µg/mL, 50 µg/mL and 75 µg/mL) are presented in Figure 9, Figure 10. The EC_50_ values (mg/mL) are presented in Table 4. Low values of EC_50_ indicate a higer effectiveness in ABTS scavenging ability.

**Table 4:** The ABTS scavenging ability (EC_50_ mg/mL) of the derivatives **6a**, **6b**, **7a_1–7_**, **7b_1–7_**.

Sample	EC_50_ mg/mL	Sample	EC_50_ mg/mL
**6a**	0.074 ± 0.0039	**6b**	0.069 ± 0.0031
**7a_1_**	0.047 ± 0.0032	**7b_1_**	0.048 ± 0.0014
**7a_2_**	0.049 ± 0.0026	**7b_2_**	0.041 ± 0.0041
**7a_3_**	0.037 ± 0.0015	**7b_3_**	0.031 ± 0.0035
**7a_4_**	0.043 ± 0.0028	**7b_4_**	0.040 ± 0.0029
**7a_5_**	0.027 ± 0.0017	**7b_5_**	0.024 ± 0.0047
**7a_6_**	0.025 ± 0.0012	**7b_6_**	0.021 ± 0.0041
**7a_7_**	0.036 ± 0.0098	**7b_7_**	0.030 ± 0.0017
**AA**	0.0055 ± 0.0024		

Data are mean ± SD (n = 3, *p*< 0.05).

From the data presented in Table 4 it is obvious that the ABTS radical scavenging ability of the benzylidenethiazolidin-4-one derivatives **7a_1–7_**, **7b_1–7_** is higher than that of the thiazolidin-4-ones **6a**, **6b**. As in the DPPH radical scavenging assay the most active compounds are the derivatives obtained by condensation of thiazolidin-4-ones with 4-hydroxy-, 4-dimethylamino- and 2-nitrobenzaldehydes. The hydroxy- (compounds **7a_5_**, **7b_5_**) and dimethylamino- (compounds **7a_6_**, **7b_6_**) analogues are about three times more active, while the nitro- (compounds **7a_7_**, **7b_7_**) are two times more active than the corresponding thiazolidin-4-ones **6a**, **6b**, but they are less active than ascorbic acid (**AA**) at the same concentration.

## 3. Experimental

### 3.1. General Procedures

The melting points were measured using a Buchi Melting Point B-540 apparatus and they are uncorrected. The FT-IR spectra were recorded on an ABB Bomen MB3000 spectrometer, over a 500–4,000 cm^−1^ range, after 32 scans at a resolution of 4 cm^−1^. The spectra processing was carried out with the Horizon MB^TM^ FTIR Software. The ^1^H-NMR and ^13^C-NMR spectra were obtained on a Bruker Avance DRX-300 spectrometer using tetramethylsilane as internal standard and DMSO-d_6_ as solvent. The chemical shifts were shown in δ values (ppm). The progress of the reaction was monitored on TLC, using pre-coated Kieselgel 60 F254 plates (Merck) and the compounds were visualized by using UV light.

### 3.2. Synthetic Procedures

#### 3.2.1. General Procedure for the Preparation of 1-[2-(1,3-Dimethylxanthin-7-yl)acetyl]-4-(4-R-phenyl)-thiosemicarbazides **5a**, **5b**

4-R-phenyl isothiocyanate (R = H, Cl; 23 mmol) was added to a solution of (1,3-dimethylxanthin-7-yl)acetyl hydrazine (7 g, 23 mmol) in mixture of 1,4-dioxane (250 mL) and DMFA (100 mL), and then heated under reflux for 6 hours. After cooling at room temperature the white solid was filtered off, dried and recrystallized from ethanol: 1,4-dioxane (3:1).

*1-[2-(1,3-Dimethylxanthin-7-yl)acetyl]-4-(phenyl)thiosemicarbazides* (**5a**). Yield 89%, m.p. 230–231 °C; FT-IR (KBr, cm^−1^): 3,238, 3,098 (-NH-), 3,012, 1,599, 1,520 (phenyl ring), 1,749 (-C=O amide), 1,680 (C=O), 1,645 (-C=N), 1,470 (-CH_2_-), 1,206 (-C-N), 1,160 (-C=S); ^1^H-NMR (DMSO-d_6_) δ in ppm: 8.09 (s, 1H, -CH=N); 8.03 (d, 1H, -NH-); 7.47, 7.35, 7.19 (dm, 5H, *Ar*-H); 5.09 (s, 2H, -CH_2_-); 3.45, 3.09 (s, 6H, 2CH_3_); 2.00 (s, 2H, -NH-C(=S)-NH-).

*1-[2-(1,3-Dimethylxanthin-7-yl)acetyl]-4-(4-chlorophenyl)thiosemicarbazides* (**5b)**. Yield 84%, m.p. 240–242 °C; FT-IR (KBr, cm^−1^): 3,285, 3,115 (-NH-), 3,040, 3,013, 1,591, 1,531 (phenyl ring), 1,693 (C=O), 1,645 (-C=N), 1,474 (-CH_2_), 1,225 (-C-N), 1,188 (-C=S), 825 (C-Cl); ^1^H-NMR (DMSO-d_6_) δ in ppm: 8.09 (s, 1H, -CH=N); 7.99 (d, 1H, -NH-); 7.51, 7.31 (d, 4H, *Ar*-H), 5.08 (s, 2H, -CH_2_-); 3.57, 3.22 (s, 6H, 2CH_3_); 2.04 (s, 2H, -NH-C(=S)-NH-).

#### 3.2.2. General Procedure for the Preparation of 2-{2-[2-(1,3-Dimethylxanthin-7-yl)acetyl]hydrazono}-3-(4-R-phenyl) Thiazolidin-4-ones (R = H, Cl) **6a**, **6b**

Chloroacetyl chloride (39 mmol) was added slowly at room temperature to a solution of 1-[2-(1,3-dimethylxanthin-7-yl)acetyl]-4-(4-R-phenyl)thiosemicarbazides **5a**, **5b** (13 mmol) in a mixture of methanol-chloroform (1:1, 250 mL). After 10 h of heating under reflux a yellow clear solution was obtained. The solvent was removed under reduced pressure and the residue was washed with cold water, (100 mL), filtered off, dried and recrystallized from ethanol 96%. The progress of the reaction was monitored by silica gel coated TLC plates.

*2-{2-[2-(1,3-Dimethylxanthin-7-yl)acethyl]hydrazono}-3-(phenyl)thiazolidin-4-one* (**6a**). Yield 52%, m.p. 239–241 °C; FT-IR (KBr, cm^−1^): 3,310, 3,201, 3,145 (-NH-), 3,048, 1,604, 1,553 (phenyl ring), 1,701 (-C=O), 1,642 (C=N), 1,471 (-CH_2_-), 1,234 (C-N), 690 (C-S); ^1^H-NMR (DMSO-d_6_) δ in ppm: 8.28 (s, 1H, -CH=N); 7.55, 7.33, 6.99 (dm, 5H, *Ar*-H); 7.01 (s, 1H, -NH-); 5.17 (s, 2H, -CH_2_-); 3.67 (s, 2H, S-CH_2_-); 3.44, 3.23 (s, 6H, 2CH_3_).

*2-{2-[2-(1,3-Dimethylxanthin-7-yl)acethyl]hydrazono}-3-(4-chlorphenyl)thiazolidin-4-one* (**6b**). Yield 50%, m.p. 248–249 °C; FT-IR (KBr, cm^−1^): 3,312, 3,223, 3,179 (-NH-), 3,051, 1,603, 1,547 (phenyl ring), 1,696 (-C=O), 1,644 (C=N), 1,466 (-CH_2_-), 1,216 (C-N), 812 (C-Cl), 695 (C-S); ^1^H-NMR ^1^H-NMR (DMSO-d_6_) δ in ppm: 7.99 (s, 1H, -CH=N ); 7.04 (s, 1H, -NH-); 7.52, 7.45 (d, 4H, *Ar*-H); 3.78 (s, 2H, S-CH_2_-); 5.08 (s, 2H, -CH_2_-); 3.41, 3.17 (s, 6H, 2CH_3_).

#### 3.2.3. General Procedure for the Preparation of 2-{2-[2-(1,3-Dimethylxanthin-7-yl)acetyl]hydrazono}-3-(4-R_1_-phenyl-5-(R_2_-benzyliden)thiazolidin-4-ones **7a_1-7_**, **7b_1-7_**

The aromatic aldehydes (79 mmol) and piperidine (79 mmol) as catalyst were added slowly to a solution of 2-{2-[2-(1,3-dimethylxanthin-7-yl)acethyl]hydrazono}-3-(4-R_1_-phenyl)thiazolidine-4-one **6a**, **6b** (79 mmol) in 1,4-dioxane (100 mL). The mixture of reaction was heated under reflux for 6 h and then the solvent was removed under reduced pressure. The crude product was precipitated in cold water, filtered off, washed with cold water and recrystallised from 1,4- dioxane.

*2-{2-[2-(1,3-Dimethylxanthin-7-yl)acethyl]hydrazono}-3-phenyl-5-(benzyliden)thiazolidin-4-one* (**7a_1_**). Yield 72%, m.p. 260–262 °C; FT-IR (KBr, cm^−1^): 3,255, 3,191 (-NH-), 3,082, 1,598, 1,547 (phenyl ring), 1,696 (-C=O), 1,641 (C=N), 1,376 (-CH_2_-), 1,229 (C-N), 680 (C-S-C); ^1^H-NMR (DMSO-d_6_) δ in ppm: 8.27 (s, 1H, -CH=N-); 7.64–6.99 (dm, 10H, *Ar*-H); 7.35 (s, 1H, C=CH-); 6.92 (s, 1H, -NH-); 5.79 (s, 2H, -CH_2_-); 3.44, 3.23 (s, 6H, 2CH_3_); ^13^C-NMR (DMSO-d_6_) δ in ppm: 173.70 (C_9_), 165.63 (C_12_), 154.44, 150.95 (C_6_, C_2_), 154.05 (C_10_), 148.41 (C_4_), 146.60 (C_7_), 142.94 (C_13_), 135.27 (C_19_), 132.19 (C_20_), 129.08 (C_22_, C_24_), 128.68 (C_15_, C_17_), 127.99 (C_16_), 127.52 (C_14_, C_18_), 124.77 (C_23_), 121.97 (C_21_, C_25_), 117.37 (C_11_), 105.77 (C_5_), 30.65 (C_8_), 29.47, 27.5 (C_1_, C_3_).

*2-{2-[2-(1,3-Dimethylxanthin-7-yl)acethyl]hydrazono}-3-phenyl-5-(4-fluorobenzyliden)thiazolidin-4-one* (**7a_2_**). Yield 70%, m.p. 263–265 °C; FT-IR (KBr, cm^−1^): 3,254, 3,138 (-NH-), 3,082, 1,599, 1,547 (phenyl ring), 1,696 (-C=O), 1,642 (C=N), 1,352 (-CH_2_-), 1,229 (C-N), 1,153 (C-F), 680 (C-S-C); ^1^H-NMR (DMSO-d_6_) δ in ppm: 8.18 (s, 1H, -CH=N-); 7.50–6.96 (dm, 9H, *Ar-*H); 7.28 (s, 1H, C=CH-); 6.89 (s, 1H, -NH-); 5.73 (s, 2H, -CH_2_-); 3.40, 3.18 (s, 6H, 2CH_3_); ^13^C-NMR (DMSO-d_6_) δ in ppm: 172.29 (C_9_), 166.36 (C_12_), 163.12 (C_16_), 155.11, 154.96 (C_6_, C_2_), 151.63 (C_10_), 148.75 (C_4_), 146.67 (C_7_), 143.07 (C_13_), 133.24 (C_19_), 129.65 (C_20_), 129.26 (C_22_, C_24_), 128.83 (C_14_, C_18_), 124.19 (C_23_), 122.71 (C_21_, C_25_), 118.05 (C_11_), 117.85 (C_15_, C_17_), 106.36 (C_5_), 31.06 (C_8_), 30.04, 28.09 (C_1_, C_3_).

*2-{2-[2-(1,3-Dimethylxanthin-7-yl)acethyl]hydrazono}-3-phenyl-5-(4-chlorobenzyliden)thiazolidin-4-one* (**7a_3_**). Yield 74%, m.p. 235–237 °C; FT-IR (KBr, cm^−1^): 3,254, 3,140 (-NH-), 3,082, 1,598, 1,547 (phenyl ring), 1,696 (-C=O), 1,641 (C=N), 1,350 (-CH_2_-), 1,229 (C-N), 804 (C-Cl), 680 (C-S-C); ^1^H-NMR (DMSO-d_6_) δ in ppm: 8.24 (s, 1H, -CH=N-); 7.55-6.99 (dm, 9H, *Ar*-H); 7.34 (s, 1H, C=CH-); 6.94 (s, 1H, -NH-); 5.77 (s, 2H, -CH_2_-); 3.42, 3.21 (s, 6H, 2CH_3_); ^13^C-NMR (DMSO-d_6_) δ in ppm: 174.41 (C_9_), 166.53 (C_12_), 154.95, 154.82 (C_6_, C_2_), 151.52 (C_10_), 148.86 (C_4_), 145.41 (C_7_), 140.76 (C_13_), 133.87 (C_19_), 134.02 (C_16_), 133.35 (C_20_), 129.61 (C_22_, C_24_), 129.26 (C_15_, C_17_), 127.88 (C_14_, C_18_), 125.47 (C_23_), 122.66 (C_21_, C_25_), 117.93 (C_11_), 106.24 (C_5_), 30.01 (C_8_), 29.03, 28.04 (C_1_, C_3_).

*2-{2-[2-(1,3-Dimethylxanthin-7-yl)acethyl]hydrazono}-3-phenyl-5-(4-bromobenzyliden)thiazolidin-4-one* (**7a_4_**). Yield 62%, m.p. 258–260 °C; FT-IR (KBr, cm^−1^): 3,254, 3,139 (-NH-), 3,081, 1,599, 1,547 (phenyl ring), 1,696 (-C=O), 1,642 (C=N), 1,352 (-CH_2_-), 1,229 (C-N), 680 (C-S-C), 660 (C-Br); ^1^H-NMR (DMSO-d_6_) δ in ppm: 8.27 (s, 1H, -CH=N-); 7.55–6.99 (dm, 9H, *Ar*-H); 7.38 (s, 1H, C=CH-); 6.92 (s, 1H, -NH-); 5.84 (s, 2H, -CH_2_-); 3.48, 3.26 (s, 6H, 2CH_3_); ^13^C-NMR (DMSO-d_6_) δ in ppm: 172.81 (C_9_), 165.63 (C_12_), 155.29, 154.44 (C_6_, C_2_), 151.22 (C_10_), 148.41 (C_4_), 146.87 (C_7_), 140.35 (C_13_), 134.63 (C_19_), 133.45 (C_20_), 131.57 (C_15_, C_17_), 129.06 (C_22_, C_24_), 128.44 (C_14_, C_18_), 125.87 (C_23_), 123.54 (C_21_, C_25_), 121.83 (C_16_), 117.38 (C_11_), 106.88 (C_5_), 31.07 (C_8_), 29.48, 27.51 (C_1_, C_3_).

*2-{2-[2-(1,3-Dimethylxanthin-7-yl)acethyl]hydrazono}-3-phenyl-5-(4-hydroxybenzyliden)thiazolidin-4-one* (**7a_5_**). Yield 78%, m.p. 243–245 °C; FT-IR (KBr, cm^−1^): 3,267 (OH), 3,253, 3,138 (-NH-), 3,081, 1,598, 1,547 (phenyl ring), 1,695 (-C=O), 1,641 (C=N), 1,350 (-CH_2_-), 1229 (C-N), 1152 (C-O), 680 (C-S-C); ^1^H-NMR (DMSO-d_6_) δ in ppm: 8.28 (s, 1H, -CH=N-); 7.75–6.99 (dm, 9H, *Ar*-H), 7.35 (s, 1H, C=CH-); 6.93 (s, 1H, -NH-); 5.79 (s, 2H, -CH_2_-); 3.32, 3.23 (s, 6H, 2CH_3_); ^13^C-NMR (DMSO-d_6_) δ in ppm: 173.33 (C_9_), 165.64 (C_12_), 158.33 (C_16_), 154.44, 154.21 (C_6_, C_2_), 150.95 (C_10_), 148.41 (C_4_), 146.47 (C_7_), 140.36 (C_13_), 132.06 (C_20_), 129.05 (C_22_, C_24_), 127.99 (C_14_, C_18_, C_19_), 125.27 (C_23_), 123.37 (C_21_, C_25_), 117.37 (C_11_), 115.79 (C_15_, C_17_), 105.77 (C_5_), 31.44 (C_8_), 29.47, 27.51 (C_1_, C_3_).

*2-{2-[2-(1,3-Dimethylxanthin-7-yl)acethyl]hydrazono}-3-phenyl-5-(4-N-dimethylaminobenzyliden)* *thiazolidin-4-one* (**7a_6_**). Yield 88%, m.p. 255–257 °C; FT-IR (KBr, cm^−1^): 3,256, 3,139 (-NH-), 1,599, 1,549 (phenyl ring), 1,699 (-C=O), 1,643 (C=N), 1,353 (-CH_2_-), 1,230 (C-N), 681 (C-S-C); ^1^H-NMR (DMSO-d_6_) δ in ppm: 8.19 (s, 1H, -CH=N-); 7.52-6.96 (dm, 9H, *Ar*-H); 7.35 (s, 1H, C=CH-); 6.92 (s, 1H, -NH-); 5.74 (s, 2H, -CH_2_-); 3.41, 3.19 (s, 6H, 2CH_3_); 2.52, 2.49 (s, 6H, 2CH_3_, N(CH_3_)_2_); ^13^C-NMR (DMSO-d_6_) δ in ppm: 173.23 (C_9_), 166.29 (C_12_), 154.95, 154.65 (C_6_, C_2_), 151.59 (C_10_), 148.79 (C_4_), 148.46 (C_16_), 147.26 (C_7_), 140.63 (C_13_), 132.07 (C_20_), 129.63 (C_14_, C_18_), 129.05 (C_22_, C_24_), 127.26 (C_23_), 124.64 (C_19_), 122.85 (C_21_, C_25_), 117.98 (C_11_), 115.62 (C_15_, C_17_), 106.33 (C_5_), 39.87 (2C, N(CH_3_)_2_), 33.45 (C_8_), 30.02, 28.07 (C_1_, C_3_).

*2-{2-[2-(1,3-Dimethylxanthin-7-yl)acethyl]hydrazono}-3-phenyl-5-(2-nitrobenzyliden)thiazolidin-4-one* (**7a_7_**). Yield 91%, m.p. 247–250 °C; FT-IR (KBr, cm^−1^): 3,255, 3,139 (-NH-), 3,082, 1,598, 1,547 (phenyl ring), 1,696 (-C=O), 1,641 (C=N), 1,499 (NO_2_), 1,350 (-CH_2_-), 1,229 (C-N), 680 (C-S-C); ^1^H-NMR (DMSO-d_6_) δ in ppm: 8.22 (s, 1H, -CH=N-); 8.21–6.99 (dm, 9H, *Ar*-H), 7.34 (s, 1H, C=CH-); 6.91 (s, 1H, -NH-); 5.76 (s, 2H, -CH_2_-); 3.42, 3.21 (s, 6H, 2CH_3_); ^13^C-NMR (DMSO-d_6_) δ in ppm: 170.61 (C_9_), 166.22 (C_12_), 155.08, 154.85 (C_6_, C_2_), 151.54 (C_10_), 148.84 (C_4_), 145.40 (C_18_), 144.45 (C_7_), 140.99 (C_13_), 133.91 (C_20_), 131.53 (C_15_), 130.12 (C_19_), 129.61 (C_22_, C_24_), 128.41 (C_16_), 127.63 (C_14_), 126.37 (C_23_), 122.72 (C_21_, C_25_), 121.11 (C_17_), 117.94 (C_11_), 106.31 (C_5_), 31.11 (C_8_), 30.01, 28.05 (C_1_, C_3_).

*2-{2-[2-(1,3-Dimethylxanthin-7-yl)acethyl]hydrazono}-3-(4-chlorophenyl)-5-(benzyliden)thiazolidin-4-one* (**7b_1_**). Yield 68%, m.p. 265–267 °C; FT-IR (KBr, cm^−1^): 3,297, 3,118 (-NH-), 2,994, 1,591, 1,545 (phenyl ring), 1,699 (-C=O), 1,639 (C=N), 1,348 (-CH_2_-), 1,214 (C-N), 777 (C-Cl), 710 (C-S-C); ^1^H-NMR (DMSO-d_6_) δ in ppm: 8.06 (s, 1H, -CH=N-); 7.51-7.31 (dm, 9H, *Ar*-H); 7.24 (s, 1H, C=CH-); 7.01 (s, 1H, -NH-); 5.17 (s, 2H, -CH_2_-); 3.32, 3.20 (s, 6H, 2CH_3_); ^13^C-NMR (DMSO-d_6_) δ in ppm: 173.73 (C_9_), 167.63 (C_12_), 156.18 (C_10_), 154.39, 150.97 (C_6_, C_2_), 148.24 (C_4_), 146.52 (C_7_), 142.81 (C_13_), 135.18 (C_19_), 132.41 (C_20_), 128.57 (C_22_, C_24_), 128.02 (C_15_, C_17_), 127.03 (C_16_), 126.87 (C_14_, C_18_), 120.84 (C_23_), 119.76 (C_21_, C_25_), 117.08 (C_11_), 106.29 (C_5_), 29.45 (C_8_), 29.01, 27.42, (C_1_, C_3_).

*2-{2-[2-(1,3-Dimethylxanthin-7-yl)acethyl]hydrazono}-3-(4-chlorophenyl)-5-(4-fluorobenzyliden) thiazolidin-4-one* (**7b_2_**). Yield 70%, m.p. 255–257 °C; FT-IR (KBr, cm^−1^): 3,229, 3,186 (-NH-), 3,000, 1,605, 1,545 (phenyl ring), 1,694 (-C=O), 1,647 (C=N), 1,344 (-CH_2_-), 1,225 (C-N), 1,149 (C-F), 759 (C-Cl), 747 (C-S-C); ^1^H-NMR (DMSO-d_6_) δ in ppm: 8.28 (s, 1H, -CH=N-); 7.62-7.36 (d, 8H, Ar-H); 7.36 (s, 1H, C=CH-); 7.02 (s, 1H, -NH-); 5.80 (s, 2H, -CH_2_-); 3.44, 3.23 (s, 6H, 2CH_3_); ^13^C-NMR (DMSO-d_6_) δ in ppm: 173.38 (C_9_), 165.31 (C_12_), 163.83 (C_16_), 155.93 (C_10_), 154.44, 150.95 (C_6_, C_2_), 148.42 (C_4_), 145.43 (C_7_), 142.84 (C_13_), 132.68 (C_20_), 130.64 (C_19_), 128.87 (C_22_, C_24_), 125.35 (C_14_, C_18_), 121.32 (C_21_, C_25_), 120.41 (C_23_), 118.88 (C_11_), 116.16 (C_15_, C_17_), 105.76 (C_5_), 30.43 (C_8_), 29.47, 27.51 (C_1_, C_3_).

*2-{2-[2-(1,3-Dimethylxanthin-7-yl)acethyl]hydrazono}-3-(4-chlorophenyl)-5-(4-chlorobenzyliden) thiazolidin-4-one* (**7b_3_**). Yield 59%, m.p. 250–252 °C; FT-IR (KBr, cm^−1^): 3,296, 3,118 (-NH-), 2,994, 1,591, 1,547 (phenyl ring), 1,699 (-C=O), 1,641 (C=N), 1,348 (-CH_2_-), 1,214 (C-N), 777 (C-Cl), 712 (C-S-C); ^1^H-NMR (DMSO-d_6_) δ in ppm: 8.28 (s, 1H, -CH=N-); 7.63-7.36 (d, 8H, *Ar-*H); 7.35 (s, 1H, C=CH-); 7.01 (s, 1H, -NH-); 5.84 (s, 2H, -CH_2_-); 3.48, 3.29 (s, 6H, 2CH_3_); ^13^C-NMR (DMSO-d_6_) δ in ppm: 173.62 (C_9_), 165.31 (C_12_), 154.66 (C_10_), 154.32, 150.87 (C_6_, C_2_), 151.78 (C_4_), 148.41 (C_7_), 142.07 (C_13_), 139.23 (C_19_), 133.35 (C_16_), 131.36 (C_20_), 129.19 (C_15_, C_17_), 128.84 (C_14_, C_18_), 125.36 (C_22_, C_24_), 123.99 (C_21_, C_25_), 121.25 (C_23_), 118.87 (C_11_), 105.76 (C_5_), 30.32 (C_8_), 29.44, 27.48 (C_1_, C_3_).

*2-{2-[2-(1,3-Dimethylxanthin-7-yl)acethyl]hydrazono}-3-(4-chlorophenyl)-5-(4-bromobenzyliden) thiazolidin-4-one* (**7b_4_**). Yield 69%, m.p. 245–247 °C; FT-IR (KBr, cm^−1^): 3,297, 3,117 (-NH-), 2,994, 1,589, 1,545 (phenyl ring), 1,699 (-C=O), 1,635 (C=N), 1,348 (-CH_2_-), 1,213 (C-N), 775 (C-Cl), 710 (C-S-C), 635 (C-Br); ^1^H-NMR (DMSO-d_6_) δ in ppm: 8.04 (s, 1H, -CH=N-); 7.49–7.33 (d, 8H, Ar-H), 7.24 (s, 1H, C=CH-); 7.03 (s, 1H, -NH-); 5.17 (s, 2H, -CH_2_-); 3.30, 3.20 (s, 6H, 2CH_3_); ^13^C-NMR (DMSO-d_6_) δ in ppm: 173.32 (C_9_), 167.61 (C_12_), 163.57 (C_16_), 154.58 (C_10_), 154.39, 154.39 (C_6_, C_2_), 150.97 (C_4_), 147.95 (C_7_), 142.04 (C_13_), 131.89 (C_20_), 130.53 (C_19_), 128.58 (C_14_, C_18_), 128.02 (C_22_, C_24_), 122.87 (C_21_, C_25_), 120.84 (C_23_), 119.77 (C_11_), 117.32 (C_15_, C_17_), 106.29 (C_5_), 33.78 (C_8_), 29.45, 27.42 (C_1_, C_3_).

*2-{2-[2-(1,3-Dimethylxanthin-7-yl)acethyl]hydrazono}-3-(4-chlorophenyl)-5-(4-hydroxybenzyliden) thiazolidin-4-one* (**7b_5_**). Yield 58%, m.p. 248–250 °C; FT-IR (KBr, cm^−1^): 3,318 (OH), 3,229, 3,187 (-NH-), 3,000, 1,607, 1,547 (phenyl ring), 1,695 (-C=O), 1,650 (C=N), 1,344 (-CH_2_-), 1,227 (C-N), 1,150 (C-O), 799 (C-Cl), 748 (C-S-C); ^1^H-NMR (DMSO-d_6_) δ in ppm: 8.28 (s, 1H,-CH=N-); 7.60-7.34 (d, 8H, *Ar*-H); 7.28 (s, 1H, C=CH-); 7.01 (s, 1H, -NH-); 5.77 (s, 2H, -CH_2_-); 3.42, 3.21 (s, 6H, 2CH_3_); ^13^C-NMR (DMSO-d_6_) δ in ppm: 173.33 (C_9_), 165.33 (C_12_), 157.43 (C_16_), 154.80 (C_10_), 151.03 (C_4_), 154.43, 150.26, (C_6_, C_2_), 148.36 (C_7_), 142.72 (C_13_), 131.82 (C_20_), 128.87 (C_14_, C_18_), 127.03 (C_19_), 125.50 (C_22_, C_24_), 124.76 (C_21_, C_25_), 123.53 (C_23_), 118.67 (C_11_), 117.08 (C_15_, C_17_), 105.79 (C_5_), 30.61 (C_8_), 29.49, 27.52 (C_1_, C_3_).

*2-{2-[2-(1,3-Dimethylxanthin-7-yl)acethyl]hydrazono}-3-(4-chlorophenyl)-5-(4-N-dimethylamino-benzyliden)thiazolidin-4-one* (**7b_6_**). Yield 65%, m.p. 258–260 °C; FT-IR (KBr, cm^−1^): 3,294, 3,118 (-NH-), 2,994, 1,590, 1,548 (phenyl ring), 1,699 (-C=O), 1,638 (C=N), 1,348 (-CH_2_-), 1,214 (C-N), 776 (C-Cl), 711 (C-S-C); ^1^H-NMR (DMSO-d_6_) δ in ppm: 8.06 (s, 1H, -CH=N-); 7.70-7.31 (d, 8H, *A*r-H), 7.16 (s, 1H, C=CH-); 7.02 (s, 1H, -NH-); 5.17 (s, 2H, -CH_2_-); 3.32, 3.20 (s, 6H, 2CH_3_); 3.05, 2.97 (s, 6H, 2CH_3_, N(CH_3_)_2_); ^13^C-NMR (DMSO-d_6_) δ in ppm: 173.54 (C_9_), 167.60 (C_12_), 154.39 (C_10_), 150.97 (C_4_), 155.28, 150.33, (C_6_, C_2_), 150.04 (C_16_), 147.95 (C_7_), 138.51 (C_13_), 131.41 (C_20_), 128.71 (C_14_, C_18_), 125.45 (C_22_, C_24_), 124.92 (C_19_), 124.12 (C_21_, C_25_), 123.11 (C_23_), 119.78 (C_11_), 113.88 (C_15_, C_17_), 105.94 (C_5_), 40.32 (2C, N(CH_3_)_2_), 30.56 (C_8_), 29.45, 27.43 (C_1_, C_3_).

*2-{2-[2-(1,3-Dimethylxanthin-7-yl)acethyl]hydrazono}-3-(4-chlorophenyl)-5-(2-nitrobenzyliden)* *thiazolidin-4-one* (**7b_7_**). Yield 75%, m.p. 235–237 °C; FT-IR (KBr, cm^−1^): 3,296, 3,118 (-NH-), 2,994, 1,591, 1,547 (phenyl ring), 1,699 (-C=O), 1,641 (C=N), 1,499 (NO_2_), 1,348 (-CH_2_-), 1,214 (C-N), 777 (C-Cl), 712 (C-S-C); ^1^H-NMR (DMSO-d_6_) δ in ppm: 8.28 (s, 1H, -CH=N-); 8.06-7.38 (d, 8H, *Ar*-H); 7.18 (s, 1H, C=CH-); 7.02 (s, 1H, -NH-); 5.80 (s, 2H, -CH_2_-); 3.44, 3.23 (s, 6H, 2CH_3_); ^13^C-NMR (DMSO-d_6_) δ in ppm: 173.96 (C_9_), 165.27 (C_12_), 154.72 (C_10_), 154.44, 148.42 (C_6_, C_2_), 150.95 (C_4_), 147.82 (C_7_), 147.42 (C_18_), 139.63 (C_13_), 137.86 (C_15_), 131.74 (C_20_), 129.64 (C_19_), 129.38 (C_16_), 128.82 (C_22_, C_24_), 127.51 (C_14_), 122.97 (C_21_, C_25_), 120.83 (C_23_), 120.6 (C_17_), 119.03 (C_11_), 105.76 (C_5_), 33.64 (C_8_), 29.46, 27.49 (C_1_, C_3_).

### 3.3. Biological Evaluation

#### 3.3.1. Antioxidant Assays

The antioxidant activity was estimated using *in vitro* tests: ferric reducing power, total antioxidant capacity, DPPH radical scavenging assay and ABTS radical scavenging ability.

#### 3.3.2. Ferric Reducing Power

The ferric reducing power of the compounds was quantified by the method described by [30] with slight modifications. phosphate buffer (1 mL, 0.2 M, pH 6.6) and potassium ferricyanide (1 mL, 1% w/v) were added in a test tube to the sample (50 µL, 100 µL, 200 µL, 300 µL) obtained from a stock solution (5 mg/mL in DMSO). The mixture was incubated at 50 °C for 20 min in a water bath and then the reaction was stopped by adding trichloroacetic acid (1 mL, 10% w/v). After centrifugation at 4,500 rpm for 15 min, the upper layer (1 mL) was collected and diluted with deionised water (1 mL) and then ferric chloride (0.2 mL, 0.1% w/v) was added. The mixture was left at room temperature for 10 min and then the absorbance was measured at 700 nm against a blank solution (DMSO mixed with the reagents). All the tests were performed in triplicate. For each sample the effective concentration (EC_50_) was calculated and the ascorbic acid (AA) was used as positive control.

#### 3.3.3. Total Antioxidant Activity

The total antioxidant activity of tested compounds was evaluated using the phosphomolybdenum method according to the procedure of [31] with minor modifications. The method is based on the reduction of Mo (VI) to Mo (V) by the tested compounds followed by the formation of a green phosphate/Mo(V) complex at acid pH. There were used different sample volumes (10, 15, 20, 30, 40, 60 µL) obtained from a stock solution (5 mg/mL in DMSO). The samples were mixed with the reagent solution (2 mL, 28 mM sodium phosphate; 4 mM ammonium molybdate; 0.6 M sulphuric acid), incubated at 95 °C for 90 min, cooled at room temperature and then centrifuged at 4,500 rpm for 15 min. The upper layer was collected and the absorbance was measured at 695 nm against a blank solution (DMSO mixed with the reagents). For each sample the effective concentration (EC_50_) was calculated and the ascorbic acid (AA) was used as positive control. All tests were performed in triplicate.

#### 3.3.4. DPPH Radical Scavenging Assay

The radical scavenging activity of the tested compounds towards the radical 1,1-diphenyl-2-picrylhydrazyl (DPPH) was measured as described by [32] with slight modifications. Various concentrations of the tested compounds (50 µL, 75 µL, 150 µL, 200 µL) from a stock solution (5 mg/mL in DMSO) were mixed with DMSO to obtain 200 μL sample, and then DPPH in methanol (2,500 μL, 0.1 mM) was added. The mixture was left for 1 hour at room temperature, in the dark, and after that the absorbance was measured at 517 nm against a blank solution. The radical scavenging capacity was calculated according to the following equation: Scavenging activity % = [(A_o_ − A_t_)/A_0_] × 100. A_0_ is the absorbance of DPPH methanol solution. A_t_ is the absorbance of the sample after 1 h. For each sample the effective concentration (EC_50_) was calculated and the ascorbic acid (AA) was used as positive control. All tests were performed in triplicate.

#### 3.3.5. ABTS Radical Scavenging Assay

The ABTS**^+^** radicals were activated by reacting of ABTS (7 mM) with ammonium persulphate (2.45 mM). The mixture was left at room temperature for 16 hours in the dark as described by [33]. The intensely coloured ABTS^+^ radical cation solution was diluted with ethanol to obtain an absorbance value of 0.7 ± 0.02 at 734 nm. Different sample volumes (5 µL, 10 µL, 20 µL, 25 µL) from a stock solution (5 mg/mL in DMSO) were mixed with DMSO to 25 µL and then ABTS solution (1975 µL) was added. After 6 min the absorbance was measured and the radical scavenging capacity was calculated according to the following equation: Scavenging activity % = [(A_o_ − A_t_)/A_0_] × 100. A_0_ is the absorbance before adding the sample. A_t_ is the absorbance after 6 min of reaction. For each sample the effective concentration (EC_50_) was calculated and the ascorbic acid (AA) was used as positive control. All tests were performed in triplicate.

#### 3.3.6. Statistical Analysis

All antioxidant assays were carried out in triplicate. Data were analyzed by an analysis of variance (ANOVA) (*p* < 0.05) and were expressed as means ± SD. The EC50 values were calculated by linear interpolation between the values registered above and below 50% activity.

## 4. Conclusions

In this study new heterocyclic compounds that combine a xanthine structure with a thiazolidine one have been synthesized. The structures of all new compounds were proven using spectral methods. The compounds were evaluated for their antioxidant activity using *in vitro* assays: ferric reducing power, total antioxidant activity, ABTS and DPPH radical scavenging ability. The all benzylidenethiazolidin-4-one derivatives **7a_1-7_**, **7b_1-7_** showed improved antioxidant effects in reference to the corresponding thiazolidin-4-ones **6a**, **6b**. The encouraging preliminary results support the antioxidant potential of the synthesized compounds and their possible applications in several diseases mediated by reactive oxygen species (ROS), including diabetes mellitus, and motivate our next research focused on their potential antidiabetic effects.
